# A Comprehensive Bibliometric Analysis of U.S. Neurosurgical Residency Programs: Evaluating Recent Research Productivity, Citation Impact, and Publication Equity

**DOI:** 10.7759/cureus.106806

**Published:** 2026-04-10

**Authors:** Maxon Bassett, Ivy B Nguyen, Sachi Patel, Leah Kunneth, Makenna Taylor, Jada E Ashford, Isha Patel, Josephine Jalkh, Nickalus Khan, L. Madison Michael II

**Affiliations:** 1 Neurosurgery, University of South Alabama College of Medicine, Mobile, USA; 2 Medicine, University of South Alabama College of Medicine, Mobile, USA; 3 Neurosurgery, The University of Tennessee Health Science Center, Memphis, USA; 4 Neurological Surgery, Semmes-Murphey Clinic, Memphis, USA

**Keywords:** academic output, h-index, neurosurgery, publication trends, residency

## Abstract

Bibliometric analyses are essential for assessing research productivity and impact in academic medicine, particularly in neurosurgery. Traditional indices, such as the h-index, provide valuable insights into scholarly output but may disproportionately reflect past productivity. The five-year h-index (ih(5)-index) addresses this limitation by focusing on recent academic contributions. This study aims to update bibliometric assessments of U.S. neurosurgical residency programs, ranking institutions based on recent research performance and examining factors influencing publication equality.

All Accreditation Council for Graduate Medical Education (ACGME)-accredited U.S. neurosurgical residency programs were included. Faculty rosters were compiled from departmental websites, and publication and citation data from 2019 to 2024 were retrieved using Scopus. Bibliometric indices, including the ih(5)-index, ig(5)-index, ie(5)-index, and i10(5)-index, were calculated. Programs were ranked based on these metrics, and regional variations were analyzed. Additionally, publication equality was assessed using Lorenz curves and Gini coefficients, with statistical analyses identifying predictors of balanced scholarly contributions.

The Western region demonstrated the highest median ih(5)-index (32.5), followed by the Northeast and Midwest (both 27), and the South (25). The top five neurosurgical programs in recent research productivity were the University of California San Francisco, Stanford Health Care, Duke University Hospital, Johns Hopkins University, and McGaw Medical Center of Northwestern University. The ih(5)-index correlated positively with total research output and negatively with publication inequality, emerging as an independent predictor of a balanced distribution of scholarly contributions.

This study provides an updated evaluation of neurosurgical research productivity, offering valuable insights for prospective residents, academic institutions, and funding bodies. The ih(5)-index serves as a key metric for assessing recent research performance and equitable scholarly contributions, ensuring that bibliometric evaluations remain relevant and informative.

## Introduction and background

Bibliometrics quantitatively assesses academic publishing using statistical methods to evaluate research productivity and scholarly impact. Citation-based indices, such as the h-index, g-index, e-index, and i10-index, are widely used to track academic influence at individual, institutional, and disciplinary levels. In medicine, these metrics often inform funding decisions, promotion, and institutional benchmarking. However, bibliometrics primarily measures patterns of publication and citation and not intrinsic scientific quality, methodological rigor, clinical impact, mentorship, or educational contributions. Citations may reflect visibility, collaboration networks, or field size as much as innovation and thus should be interpreted as complementary indicators rather than definitive measures of excellence.

Neurosurgery has increasingly adopted bibliometric analyses to assess research output among individuals, residency programs, and departments [1]. Traditional cumulative indices, such as the h-index, provide valuable insights but may disproportionately weight seniority and historical productivity, potentially obscuring current academic momentum. Ponce et al. [2] evaluated U.S. and Canadian neurosurgical programs but included non-neurosurgical publications, complicating field-specific assessments. Similarly, Khan et al. [3] ranked neurosurgeons using cumulative h-indices but faced limitations in reflecting recent research activity. To address these concerns, Taylor et al. [4] introduced the five-year h-index (ih(5)-index), a temporally restricted measure focusing on recent publication performance, offering a more sensitive assessment of contemporary productivity.

Database selection is critical in bibliometric studies, particularly when equity and reproducibility are central considerations. We utilized Scopus due to its broad journal coverage across medical and surgical subspecialties, structured author identification system (Scopus Author ID), and application programming interface (API) capabilities that enhance transparency and reproducibility. Compared with databases lacking standardized author disambiguation, Scopus facilitates more reliable attribution of publications and citations, which is especially important when evaluating multi-institutional and collaborative specialties such as neurosurgery.

Despite prior advancements, neurosurgery continues to evolve, necessitating updated and methodologically rigorous bibliometric evaluations. Previous studies have primarily focused on resident publication trends; however, a comprehensive assessment should incorporate faculty and department-wide output to better characterize institutional academic environments [5]. We pre-specified the following hypotheses: (i) that five-year indices would demonstrate meaningful variability across programs independent of cumulative h-indices and (ii) that temporally restricted metrics would more accurately reflect contemporary academic momentum than lifetime measures alone.

This study updates bibliometric analyses of U.S. neurosurgical residency programs using current methods and temporally sensitive indices. By revisiting the ih(5)-index and related metrics, we aim to: (i) provide prospective residents with data to inform program selection, (ii) encourage departments to sustain or enhance recent scholarly productivity, and (iii) offer institutions updated insights for resource allocation, faculty development, and academic planning. By explicitly acknowledging the strengths and limits of citation-based metrics and justifying methodological choices, this work builds upon prior studies while promoting a more transparent and contextually grounded evaluation of academic performance in neurosurgery.

## Review

Materials & methods

A comprehensive list of accredited neurological surgery residency training programs for the year 2024 was assembled based on information from the Accreditation Council for Graduate Medical Education (ACGME, https://www.acgme.org/specialties/neurological-surgery/overview/). Faculty names were extracted from departmental websites, specifically noting departmental roles. Two different experimental groups were produced from each department. One group included only neurosurgeons listed as either core or affiliated staff; this group only includes people who achieved a Doctor of Medicine (M.D.), Bachelor of Medicine, Bachelor of Surgery (MBBS), or Doctor of Osteopathic Medicine (D.O.) title. This group is noted as “Neurosurgeon-Specific Analysis”. The other group included all staff found on a department’s neurosurgical faculty; this includes research staff, joint faculty, and adjunct faculty but excludes courtesy and emeritus staff. This group is noted as “All Faculty”.

Bibliometric Analysis

Each neurosurgical institution was treated as a single entity, where the career total and five-year academic output of each faculty member, quantified through publications and citations, was aggregated to derive various bibliometric metrics outlined below. A systematic protocol was established for gathering publication and citation information to ensure the integrity and accuracy of the evaluation concerning each institution’s scholarly contributions to neurosurgery over the specified period.

For an article to contribute to an institution's bibliometric profile, it was required that at least one neurosurgeon affiliated with that institution be listed as an author. For instance, if a paper had 10 authors, including five neurologists, three neuroradiologists, and one neurosurgeon from a specific institution, it would be counted toward that institution’s output. Conversely, papers authored solely by neurologists or other specialties without a neurosurgeon were excluded from consideration.

Once a complete faculty roster was established for each institution, publication and citation data were retrieved using Scopus (Elsevier, www.scopus.com). Scopus API was utilized to collect a full summary of faculty data. For each author, a full list of all publications and citations received was collected as well as summary data including the current H index. In cases where multiple authors from the same institution contributed to a single paper, a specific authorship assignment algorithm was applied: publications were credited in the order of authorship priority (first, second, last, etc.). If a publication involved authors from multiple institutions, each institution was credited accordingly, but no more than one author per institution was included in the count.

Definition of Metrics

Using Scopus, the total number of publications and citations for each institution entirely and from 2019 to 2024 was identified and entered into a Microsoft Excel spreadsheet, sorted by citation count in descending order. The following bibliometric indices were computed for each institution (noted as “(5)” to signify the five-year period).

ih(5)-index: This index represents the count of publications (h) that have received at least h citations. This metric reflects the intersection of publication and citation counts [6].

ig(5)-index: This index represents the total number of publications (g) that collectively have received at least g² citations, complementing the ih(5)-index by emphasizing highly cited works [7].

ie(5)-index: This index assesses the excess citations from the papers that constitute the ih(5)-index, providing insight into citation impact beyond the minimum threshold required for the ih(5)-index [8].

i10(5)-index: Initially developed by Google Scholar, this index counts the number of articles produced by an institution that received 10 or more citations within the designated five-year timeframe [4].

Ranking of Programs

All ACGME-accredited U.S. neurosurgical training programs were ranked based on the defined bibliometric metrics, total publication counts, citation totals, five-year publications, five-year citations, summed H-index and Gini coefficients for both publications and citations.

Publication Equality

To evaluate academic equality among faculty members within each department, Lorenz curves and Gini coefficients were calculated for the publications and citations. The Lorenz curve plots cumulative percentages of authors against cumulative percentages of publications or citations, with equal contributions yielding a 45° line of equality. The curve was constructed using publication data from neurosurgeons affiliated with each department during the specified timeframe. The Gini coefficient, a mathematical representation of inequality in publication contributions, was derived from the Lorenz curve, where a Gini coefficient of 0 signifies equal contributions and a value of 1 indicates total disparity.

Statistical Analysis

All statistical analyses were conducted using R version 4.3.1 (R Foundation for Statistical Computing, Vienna, Austria). Following the calculation of institution-specific metrics and Gini coefficients, pooled descriptive statistics were generated for the programs. Metrics were then compared based on the geographic classification of institutions as defined by the U.S. Census Bureau. Bivariate correlation analyses were conducted to explore relationships among various indices and to assess potential multicollinearity.

We also aimed to determine whether any of the bibliometric indices could predict intradepartmental publication equality, operationalized as a Gini coefficient for publications (Ginipub) ≤ 0.5. Univariate analyses were performed comparing institutions with Ginipub ≤ 0.5 against those with higher values, followed by multivariable logistic regression accounting for multicollinearity among metrics.

Results

Bibliometric Analysis and Regional Comparisons

The national and regional bibliometric characteristics of neurosurgical training programs with ACGME accreditation in the US are listed in Table 1, including the median number of faculty, median number of total publications, median number of publications in the past five years, median number of total citations, median number of citations in the past five years, median ih(5), ig(5), ie(5), i10(5) values, and summed total ih(5) value. The full analysis including research faculty can be found in Table 2.

**Table 1:** National and Regional Characteristics of Neurosurgery Training Programs: Neurosurgeon-Specific Analysis NS: Neurosurgeon-Specific Analysis; Yr: Year; Pub: Publications

Characteristic	Total	Midwest	Northeast	South	West
Programs Included	n=115 (100%)	n=27 (23%)	n=33 (29%)	n=37 (32%)	n=18 (16%)
Median Faculty NS	17	14.5	18	16	20
Median Total Pub NS	1141	1104.5	1077	1060	1324.5
Median 5 Yr Publications NS	440	401.5	469	359	502
Median Total Citations NS	39116	38293	33981	34625	49568
Median 5 Yr Citations NS	5200	4477	4892	4551	6878.5
h-index					
Median ih(5)-Index NS	28	27	27	25	32.5
Summed ih(5) NS	837	727	942	1067	610
Median ig(5)-Index NS	7	7.5	7	6	8.5
Median ie(5)-Index NS	33	34.5	30	28	39.5
Median i10(5)-Index NS	129	107	115	133	160

**Table 2:** National and Regional Characteristics of Neurosurgery Training Programs: All Faculty Yr: Year; Pub: Publications

Characteristic	Total	Midwest	Northeast	South	West
Programs Included	n=115	n=27	n=33	n=37	n=18
Mean Faculty	24	21	22	25	30.5
Mean Total Pub	1739	1382	1770	1708	2025
Mean 5 Yr Publications	739	711	1018	409	819
Mean Total Citations	61434	51342	65182	57687	97217
Mean 5 Yr Citations	8085	9489	11161	4602	6681
h-index					
Mean ih(5)-Index	41	33	44	39	42.5
Summed ih(5)	4705	1035	1351	1484	835
Mean ig(5)-Index	9.5	9	10	9	10.5
Mean ie(5)-Index	47.5	43	52	41	53
Mean i10(5)-Index	208.5	124	213	204	240

When considering only neurosurgeons involved in research, the Western region of the United States was found to be the most academically productive with an ih(5)-index of 32.5 with a median faculty of 20. The West was followed by the Northeast and Midwest, both of which with an ih(5)-index of 27 with a median faculty of 18 and 14.5, respectively. These were followed by the South, which possessed an ih(5)-index of 25 with a median number of faculty of 16.

All bibliometric indices, ih(5), summed h, ig(5), ie(5), and i10(5), were found to be significantly positively correlated with the number of faculty, total publications, and total citations and negatively correlated with publication and citation Gini coefficients (Table 3). The full analysis including research faculty can be found in Table 4.

**Table 3:** Spearman’s Coefficient and Significance Values for Bibliometric Indices: Neurosurgeon-Specific Analysis Mann–Whitney U statistical analysis used for p-values The p-value represents the probability of observing the study results under the assumption that the null hypothesis is true. A p-value <0.05 was considered statistically significant. A Spearman's coefficient measures how similarly two variables are ordered A Spearman's coefficient of +1 signifies whoever is first in "X" is also first in "Y", the inverse is true when the Spearman's coefficient is -1 Spearman's coefficient=1−[(6∑d_i_​^2^​)/(n(n^2^−1))], where d_i_ is the difference between the ranks of paired observations and n is the number of observations A Gini coefficient is a measure of inequality within a distribution A Gini coefficient > 0.5 signifies that a value is being driven by a small percent of the population G=∑^n^_i=1_​ * ∑^n^_j=1_​ * ∣x_i_−x_j_∣​/(2n^2^x) Coeff: Coefficient

Metric	Number of Faculty	Total Publications	Total Citations	Gini Coeff Publication	Gini Coeff Citations
h-Index					
ih(5)-Index	0.69; p < 0.001	0.86; p < 0.001	0.97; p < 0.001	-0.40; p < 0.001	-0.41; p < 0.001
Summed ih(5)	0.85; p < 0.001	0.83; p < 0.001	0.83; p < 0.001	-0.26; p < 0.001	-0.28; p < 0.001
ig(5)-Index	0.68; p < 0.001	0.84; p < 0.001	0.97; p < 0.001	-0.42; p < 0.001	-0.30; p < 0.001
ie(5)-Index	0.64; p < 0.001	0.81; p < 0.001	0.85; p < 0.001	-0.33; p < 0.001	-0.20; p < 0.001
i10(5)-Index	0.73; p < 0.001	0.92; p < 0.001	0.92; p < 0.001	-0.40; p < 0.001	-0.41; p < 0.001

**Table 4:** Spearman’s Coefficient and Significance Values for Bibliometric Indices: All Faculty Mann–Whitney U statistical analysis used for p-values The p-value represents the probability of observing the study results under the assumption that the null hypothesis is true. A p-value <0.05 was considered statistically significant. A Spearman's coefficient measures how similarly two variables are ordered A Spearman's coefficient of +1 signifies that whoever is first in "X" is also first in "Y", the inverse is true when the Spearman's coefficient is -1 Spearman's Coefficient=1−[(6∑di​2​)/(n(n2−1))], where di is the difference between the ranks of paired observations and n is the number of observations A Gini coefficient is a measure of inequality within a distribution A Gini coefficient > 0.5 signifies a value is being driven by a small percent of the population G=∑ni=1​ * ∑nj=1​ * ∣xi−xj∣​/(2n2x) Coeff: Coefficient

Metric	Number of Faculty	Total Publications	Total Citations	Gini Coeff Publication	Gini Coeff Citations
h-Index					
ih(5)-Index	0.66; p < 0.001	0.91; p < 0.001	0.96; p < 0.001	-0.41; p < 0.001	-0.45; p < 0.001
Summed ih(5)	0.84; p < 0.001	0.84; p < 0.001	0.84; p < 0.001	-0.27; p < 0.001	-0.3; p = 0.002
ig(5)-Index	0.67; p < 0.001	0.89; p < 0.001	0.96; p < 0.001	-0.43; p < 0.001	-0.34; p = 0.003
ie(5)-Index	0.62; p < 0.001	0.78; p < 0.001	0.93; p < 0.001	-0.36; p < 0.001	-0.22; p = 0.003
i10(5)-Index	0.7; p < 0.001	0.9; p < 0.001	0.95; p < 0.001	-0.44; p < 0.001	-0.44; p < 0.001

Institutional Rank

Rankings are based on obtainable data for all US neurosurgical departments according to the ih(5)-index. The top five most academically productive neurosurgical programs when considering neurosurgeons specifically were the University of California San Francisco, Stanford Health Care, Duke University Hospital, Johns Hopkins University, and McGaw Medical Center of Northwestern University (Table 5). The full analysis including research faculty can be found in Table 6.

**Table 5:** Institutional Rankings Based on ih(5): Neurosurgeon-Specific Analysis NS: Neurosurgeon-Specific Analysis; Yr: Year

Rank	Author Affiliation	Mean ih(5) NS	Mean Number of Faculty NS	Mean 5 Yr Citations NS	Mean 5 Yr Publications NS
1	University of California (San Francisco)	55	36	33369	1467
2	Stanford Health Care	54	34	24734	1216
3	Duke University Hospital	54	31	27972	1679
4	Johns Hopkins University	53	24	23508	2265
5	McGaw Medical Center of Northwestern University	52	23	19136	936
6	Yale-New Haven Medical Center	51	22	25332	1049
7	Baylor College of Medicine	51	28	18475	1053
8	Massachusetts General Hospital	50	24	29445	1391
9	University of Texas Southwestern Medical Center	49	26	15888	972
10	Brigham and Women's Hospital/Children's Hospital	49	24	14835	1080
11	UCLA David Geffen School of Medicine/UCLA Medical Center	48	22	15182	901
12	University of Washington	47	26	17971	819
13	Mayo Clinic College of Medicine and Science (Rochester)	46	21	18558	2291
14	Barrow Neurological Institute at St Joseph's Hospital and Medical Center	46	26	12652	1331
15	Cedars-Sinai Medical Center	46	14	7635	379
16	New York Presbyterian Hospital (Columbia Campus)	46	21	12218	834
17	Ohio State University Hospital	44	25	15756	1101
18	University of Texas Health Science Center at Houston	44	20	13916	1315
19	University at Buffalo	43	10	12181	946
20	Westchester Medical Center	43	13	10891	851
21	Icahn School of Medicine at Mount Sinai	42	30	28066	1209
22	University of Tennessee	42	21	10386	826
23	NYU Grossman School of Medicine	41	26	18192	1409
24	Washington University/B-JH/SLCH Consortium	41	25	12944	900
25	University of Florida	41	20	13779	488
26	Case Western Reserve University/University Hospitals Cleveland Medical Center	41	14	10861	398
27	University of Oklahoma Health Sciences Center	41	16	9489	711
28	University of Pennsylvania Health System	40	25	17655	1339
29	UPMC Medical Education	40	26	15627	1284
30	University of Alabama Medical Center	40	21	11430	766
31	University of Virginia Medical Center	40	16	10129	1077
32	Vanderbilt University Medical Center	40	19	11520	1069
33	Sidney Kimmel Medical College at Thomas Jefferson University/TJUH	39	19	11617	1005
34	University of California (Irvine)	39	12	8266	1100
35	New York Presbyterian Hospital (Cornell Campus)	38	21	10657	938
36	University of Wisconsin Hospitals and Clinics	38	24	19156	525
37	University of Southern California/LAC+USC Medical Center	38	20	9040	978
38	West Virginia University	36	14	5403	387
39	University of Miami/Jackson Health System	35	23	12396	1118
40	Henry Ford Hospital	34	18	4578	405
41	Mayo Clinic College of Medicine and Science (Jacksonville)	33	15	9574	884
42	Oregon Health & Science University	33	20	6122	493
43	Clinical Center at the National Institutes of Health	33	7	4602	389
44	University of Chicago	33	10	4376	291
45	Brown University	32	21	7657	789
46	University of Utah Health	32	24	8908	1024
47	Medical College of Wisconsin Affiliated Hospitals	32	25	7203	683
48	Medical University of South Carolina	32	19	5491	516
49	MedStar Health/Georgetown University Hospital	31	17	7250	852
50	Rush University Medical Center	31	13	5222	408
51	University of Iowa Hospitals and Clinics	30	16	8864	691
52	University of California Davis Health	30	15	4643	265
53	University of New Mexico School of Medicine	30	12	4548	408
54	Wake Forest University School of Medicine	29	14	5817	429
55	Ascension Providence/MSUCHM	29	15	7288	470
56	University of Kentucky College of Medicine	29	13	2919	256
57	University of Cincinnati Medical Center/College of Medicine	28	12	4102	443
58	Rutgers Health/New Jersey Medical School	28	7	3803	335
59	Penn State Milton S Hershey Medical Center	27	22	4892	366
60	Montefiore Medical Center/Albert Einstein College of Medicine	27	14	3438	434
61	University of California (San Diego) Medical Center	26	20	4733	511
62	University of Minnesota	26	14	3704	398
63	Geisinger Health System	26	18	3862	469
64	Philadelphia College of Osteopathic Medicine	26	9	3186	342
65	University of Missouri-Columbia	26	8	3618	231
66	University of Michigan Health System	25	17	9802	625
67	Cleveland Clinic Foundation	25	21	7187	802
68	Tulane University/Ochsner Clinic Foundation	25	17	4113	416
69	University of Louisville School of Medicine	25	13	3726	361
70	Allegheny Health Network Medical Education Consortium (AGH)	24	22	5291	470
71	Methodist Hospital (Houston)	24	19	2394	277
72	University of Colorado	23	22	3794	367
73	Carolinas Medical Center	23	16	2514	326
74	Emory University School of Medicine	22	21	4551	491
75	Tufts Medical Center	22	8	2438	211
76	Indiana University School of Medicine	21	17	1801	230
77	Mayo Clinic College of Medicine and Science (Arizona)	21	10	2236	421
78	University of Massachusetts Medical School	21	8	1184	91
79	University of Nebraska Medical Center College of Medicine	21	11	1630	162
80	University of Maryland	20	11	5888	444
81	Texas A&M College of Medicine-Scott and White Medical Center	20	14	1431	151
82	University of Illinois College of Medicine at Chicago	20	9	2003	276
83	Zucker School of Medicine at Hofstra/Northwell	19	22	5363	542
84	University of South Florida Morsani	19	14	3125	330
85	Virginia Commonwealth University Health System	19	17	2967	273
86	George Washington University	19	20	2507	255
87	University of Arkansas for Medical Sciences (UAMS) College of Medicine	19	11	1415	178
88	Spectrum Health/Michigan State University	18	16	1549	201
89	Loyola University Medical Center	18	5	1469	227
90	Medical College of Georgia	18	12	1018	89
91	University of Kansas School of Medicine	18	17	1673	148
92	University of Rochester	18	18	1510	249
93	University of Texas at Austin Dell Medical School	17	21	1841	190
94	University of Texas Medical Branch Hospitals	17	7	963	100
95	Carilion Clinic-Virginia Tech Carilion School of Medicine	17	8	2345	92
96	Dartmouth-Hitchcock/Mary Hitchcock Memorial Hospital	17	9	983	122
97	University of North Carolina Hospitals	16	10	2018	201
98	SUNY Upstate Medical University	16	10	966	155
99	Loma Linda University Health Education Consortium	15	11	2019	180
100	Beaumont Health (Royal Oak and Dearborn)	15	13	1626	148
101	Louisiana State University (Shreveport)	15	11	1623	354
102	Albany Medical Center	15	12	925	179
103	St Louis University School of Medicine	15	8	826	187
104	Stony Brook Medicine	14	9	1407	86
105	University of Arizona College of Medicine-Tucson	14	11	1601	107
106	University of Illinois College of Medicine at Peoria	13	7	452	54
107	Riverside University Health System	12	9	565	47
108	Temple University Hospital	12	11	760	76
109	Beth Israel Deaconess Medical Center	11	9	521	86
110	Inova Fairfax Medical Campus	11	11	543	44
111	Louisiana State University	9	12	571	35
112	University of Mississippi Medical Center	8	10	487	80
113	University of Connecticut School of Medicine	8	8	341	53
114	University of Vermont Medical Center	7	6	218	58
115	Southern Illinois University School of Medicine	5	6	97	38

**Table 6:** Institutional Rankings Based on ih(5): All Faculty Yr: Year

Rank	Author Affiliation	i(h)5	Number of Faculty	5 Yr Citations	5 Yr Publications
1	McGaw Medical Center of Northwestern University	118	62	77098	3189
2	University of California (San Francisco)	102	86	67841	3477
3	Baylor College of Medicine	87	52	39984	2388
4	Duke University Hospital	85	65	59379	3240
5	Stanford Health Care	82	72	62221	3019
6	University of Pennsylvania Health System	82	58	43109	2856
7	NYU Grossman School of Medicine	81	66	36456	2851
8	Washington University/B-JH/SLCH Consortium	77	48	32859	1864
9	Icahn School of Medicine at Mount Sinai	75	52	61408	2955
10	Yale-New Haven Medical Center	75	35	33987	1393
11	University of Miami/Jackson Health System	74	55	33331	2526
12	University of Michigan Health System	73	54	39726	2608
13	Massachusetts General Hospital	72	26	29445	1391
14	University of Florida	68	50	31520	1278
15	University of Washington	68	47	26551	1277
16	UPMC Medical Education	64	54	27910	2433
17	University of Texas Health Science Center at Houston	63	50	23733	2032
18	University of Texas Southwestern Medical Center	62	46	24271	1582
19	Brigham and Women's Hospital/Children's Hospital	61	41	22393	1549
20	Ohio State University Hospital	61	37	22059	1542
21	University of Maryland	58	44	22730	1591
22	Johns Hopkins University	56	27	23508	2265
23	Sidney Kimmel Medical College at Thomas Jefferson University/TJUH	56	45	22045	1763
24	Barrow Neurological Institute at St Joseph's Hospital and Medical Center	55	44	19670	1949
25	University of Connecticut School of Medicine	53	17	9382	447
26	UCLA David Geffen School of Medicine/UCLA Medical Center	52	24	16562	983
27	Brown University	51	52	15653	1653
28	University of Iowa Hospitals and Clinics	51	42	19278	1391
29	University of Utah Health	51	45	19875	1720
30	Medical College of Wisconsin Affiliated Hospitals	50	56	13692	1210
31	New York Presbyterian Hospital (Cornell Campus)	50	33	14948	1331
32	Cedars-Sinai Medical Center	49	20	9679	535
33	University of Wisconsin Hospitals and Clinics	49	37	25337	719
34	Loma Linda University Health Education Consortium	48	28	13240	675
35	Mayo Clinic College of Medicine and Science (Rochester)	48	22	19442	2400
36	University of Alabama Medical Center	48	28	13607	912
37	Zucker School of Medicine at Hofstra/Northwell	48	43	13930	1405
38	Allegheny Health Network Medical Education Consortium (AGH)	47	46	11161	1065
39	New York Presbyterian Hospital (Columbia Campus)	46	21	12218	834
40	Methodist Hospital (Houston)	45	49	9067	700
41	Westchester Medical Center	45	23	11885	958
42	Penn State Milton S Hershey Medical Center	44	39	10299	737
43	University at Buffalo	44	22	13055	1095
44	University of South Florida Morsani	44	35	10938	922
45	Wake Forest University School of Medicine	44	22	8800	656
46	Case Western Reserve University/University Hospitals Cleveland Medical Center	43	24	13196	581
47	Tulane University/Ochsner Clinic Foundation	43	34	10984	1364
48	University of Southern California/LAC+USC Medical Center	43	32	13146	1474
49	University of Tennessee	43	21	10386	826
50	Cleveland Clinic Foundation	42	35	11978	1337
51	Mayo Clinic College of Medicine and Science (Jacksonville)	42	19	12127	1120
52	Oregon Health & Science University	42	38	9612	772
53	University of Oklahoma Health Sciences Center	41	16	9489	711
54	University of California (Irvine)	40	35	8707	1114
55	University of Virginia Medical Center	40	16	10129	1077
56	Vanderbilt University Medical Center	40	21	11561	1073
57	Emory University School of Medicine	39	48	8993	1018
58	Medical University of South Carolina	39	27	7015	670
59	University of Colorado	39	56	8033	743
60	University of Cincinnati Medical Center/College of Medicine	38	25	7230	811
61	West Virginia University	38	20	5986	436
62	Henry Ford Hospital	36	21	4832	427
63	Rutgers Health/New Jersey Medical School	35	14	6112	550
64	University of California Davis Health	35	29	6640	376
65	University of Minnesota	35	20	5443	537
66	Clinical Center at the National Institutes of Health	33	7	4602	389
67	Medical College of Georgia	33	25	4018	256
68	University of Arizona College of Medicine-Tucson	33	28	7447	412
69	University of California (San Diego) Medical Center	33	25	5916	639
70	University of Chicago	33	10	4376	291
71	University of Louisville School of Medicine	33	17	4873	472
72	University of Kentucky College of Medicine	32	30	4043	362
73	Ascension Providence/MSUCHM	31	17	7774	501
74	MedStar Health/Georgetown University Hospital	31	28	7250	852
75	Rush University Medical Center	31	14	5227	409
76	Spectrum Health/Michigan State University	31	35	9489	711
77	Virginia Commonwealth University Health System	31	28	4886	449
78	University of New Mexico School of Medicine	30	16	4548	408
79	Geisinger Health System	29	20	4291	521
80	Indiana University School of Medicine	29	24	3575	313
81	University of Massachusetts Medical School	29	10	2071	148
82	George Washington University	27	35	4172	429
83	Montefiore Medical Center/Albert Einstein College of Medicine	27	14	3438	434
84	University of North Carolina Hospitals	27	21	3502	353
85	Philadelphia College of Osteopathic Medicine	26	9	3186	342
86	University of Missouri-Columbia	26	9	3618	231
87	University of Texas at Austin Dell Medical School	25	30	2697	277
88	Carolinas Medical Center	24	18	2671	346
89	University of Nebraska Medical Center College of Medicine	23	15	2142	238
90	Stony Brook Medicine	22	14	2188	134
91	Tufts Medical Center	22	8	2438	211
92	University of Arkansas for Medical Sciences (UAMS) College of Medicine	22	15	1805	253
93	Beaumont Health (Royal Oak and Dearborn)	21	18	2251	205
94	Mayo Clinic College of Medicine and Science (Arizona)	21	10	2236	421
95	Texas A&M College of Medicine-Scott and White Medical Center	20	15	1431	151
96	University of Illinois College of Medicine at Chicago	20	9	2003	276
97	University of Texas Medical Branch Hospitals	20	12	1258	129
98	Carilion Clinic-Virginia Tech Carilion School of Medicine	19	11	2774	127
99	Louisiana State University (Shreveport)	19	14	2066	450
100	Loyola University Medical Center	18	5	1469	227
101	University of Kansas School of Medicine	18	18	1673	148
102	University of Mississippi Medical Center	18	23	1071	177
103	University of Rochester	18	19	1524	257
104	Dartmouth-Hitchcock/Mary Hitchcock Memorial Hospital	17	9	983	122
105	Louisiana State University	17	41	1226	96
106	SUNY Upstate Medical University	16	12	966	155
107	Albany Medical Center	15	12	925	179
108	St Louis University School of Medicine	15	10	826	189
109	University of Illinois College of Medicine at Peoria	13	7	452	54
110	Inova Fairfax Medical Campus	12	16	664	84
111	Riverside University Health System	12	11	569	49
112	Temple University Hospital	12	11	760	76
113	Beth Israel Deaconess Medical Center	11	9	521	86
114	University of Vermont Medical Center	7	6	218	58
115	Southern Illinois University School of Medicine	5	6	97	38

Predicting Intradepartmental Publishing Equality

Sixty (52%) of the 115 programs achieved the target GiniPub when only neurosurgeons were considered. When institutions were separated by GiniPub ≤ 0.5, we observed a statistically significant difference between all variables except for median faculty, summed ih(5), and median ig(5)-index when considering only neurosurgeons (Table 7). The full analysis including research faculty can be found in Table 8.

**Table 7:** Gini Coefficient Analysis: Neurosurgeon-Specific Analysis Mann–Whitney U statistical analysis used for p-values NS: Neurosurgeon-Specific Analysis; Yr: year A Gini coefficient is a measure of inequality within a distribution A Gini coefficient > 0.5 signifies a value is being driven by a small percent of the population G=∑ni=1 * ∑nj=1 * ∣xi−xj∣/(2n2x) The Mann–Whitney U statistic is a nonparametric measure used to compare differences between two independent groups based on ranked data. Smaller U values indicate greater separation between groups, with significance determined by the corresponding p-value.

Characteristic	Gini Coefficient ≤ 0.5	Gini Coefficient > 0.5	p-Value (U value)
Median Faculty NS	15	16	0.55 (1543)
Median Total Pub NS	1248	1643	0.03 (1399)
Median 5 Yr Publications NS	511	562	0.03 (1263)
Median Total Citations NS	41058	46058	0.001 (1062)
Median 5 Yr Citations NS	5133	6474	0.001 (1091)
h-Index			
Median ih(5) NS	24	31	0.001 (1059)
Summed ih(5) NS	1569	1635	0.11 (1364)
Median ig(5)-Index NS	6	7	0.21 (1427)
Median ie(5)-Index NS	29	35	0.001 (1064)
Median i10(5)-Index NS	119	146	0.001 (1075)

**Table 8:** Gini Coefficient Analysis: All Faculty Mann–Whitney U statistical analysis used for p-values Yr: year A Gini coefficient is a measure of inequality within a distribution A Gini coefficient > 0.5 signifies a value is being driven by a small percent of the population G=∑ni=1 * ∑nj=1 * ∣xi−xj∣/(2n2x) The Mann–Whitney U statistic is a nonparametric measure used to compare differences between two independent groups based on ranked data. Smaller U values indicate greater separation between groups, with significance determined by the corresponding p-value.

Characteristic	Gini Coefficient ≤ 0.5	Gini Coefficient > 0.5	p-Value (U value)
Mean Faculty	22	36	0.004 (1107)
Mean Total Pub	2107	3107	0.001 (1050)
Mean 5 Yr Publications	771	1049	0.001 (1044)
Mean Total Citations	85465	139993	0.001 (1067)
Mean 5 Yr Citations	10290	15307	0.001 (1060)
h-index			
Mean ih(5)-index	35	45	0.001 (1089)
Summed ih(5)	1618	3125	0.001 (1058)
Mean ig(5)-index	9	11	0.001 (1071)
Mean ie(5)-index	42	58	0.001 (1049)
Mean i10(5)-index	234	330	0.001 (1066)

After adjustment for multicollinearity, the multivariable logistic regression analysis identified the ih(5)-index as an independent predictor of a GiniPub ≤ 0.5 (Table 9). The logistic regression was adequately calibrated based on a nonsignificant Hosmer-Lemeshow goodness-of-fit p-value of 0.36, and the model had good discrimination based on area under the receiver operator characteristic curve of 0.55. The full analysis including research faculty can be found in Table 10.

**Table 9:** Multivariable Logistic Regression Analysis: Neurosurgeon-Specific Analysis Mann–Whitney U statistical analysis used for p-values The adjusted odds ratio represents the association between a predictor and a binary outcome after controlling for other variables in the model. OR >1 indicates increased odds of the outcome, whereas an OR <1 indicates decreased odds 95% confidence interval indicates the range within which the true population effect is expected to lie with 95% confidence, intervals that do not include 1.0 are considered statistically significant. The p-value reflects the probability of observing the results under the null hypothesis, with values <0.05 considered statistically significant.

Variable	Adjusted OR	95% CI	p-Value
ih(5)-Index	1.24	1.04-1.28	0.02
Summed h-Index	0.99	0.99-1.01	0.55
i10-Index	0.99	0.99-1.01	0.58

**Table 10:** Multivariable Logistic Regression Analysis: All Faculty Mann–Whitney U statistical analysis used for p-values The adjusted odds ratio represents the association between a predictor and a binary outcome after controlling for other variables in the model. OR >1 indicates increased odds of the outcome, whereas an OR <1 indicates decreased odds 95% confidence interval indicates the range within which the true population effect is expected to lie with 95% confidence, intervals that do not include 1.0 are considered statistically significant. The p-value reflects the probability of observing the results under the null hypothesis, with values <0.05 considered statistically significant.

Variable	Adjusted OR	95% CI	p-Value
ih(5)-Index	1.26	1.04-1.37	0.02
Summed h-Index	1.02	0.98-1.03	0.31
i10-Index	0.99	0.99-1.01	0.61

Discussion

Evaluating academic productivity in neurosurgery is important for assessing program standing, informing departmental strategy, and attracting faculty and trainees. In an increasingly competitive academic environment, bibliometric indices provide accessible and standardized measures of scholarly output. The h-index remains one of the most widely used metrics because it integrates productivity and citation impact; however, it inherently favors senior faculty and programs with long-standing publication histories due to cumulative citation accrual and time-dependent growth [9]. Consequently, the h-index may underrepresent the contributions of early-career faculty and may not fully capture recent changes in institutional research momentum. The ih(5)-index, which measures productivity within the past five years, addresses this limitation by emphasizing contemporary scholarly activity and reducing the influence of historically prolific individuals whose output may not reflect current departmental dynamics [10-13].

Beyond addressing citation lag, time-restricted metrics such as the ih(5)-index may also help contextualize structural disparities embedded in cumulative bibliometric measures. Prior literature has reported that women in academic medicine often demonstrate lower cumulative h-indices despite similar productivity when adjusted for career duration, a pattern attributed to multiple factors including career interruptions, differences in mentorship access, and historical disparities in funding and authorship opportunities [10]. By restricting analysis to a defined five-year window, the ih(5)-index partially normalizes for career duration and emphasizes recent scholarly engagement. Additionally, limiting the time frame may improve bibliometric accuracy for investigators who publish under multiple name variations or whose publication records are fragmented across databases [14]. While the present study did not directly measure gender representation or authorship practices, these considerations provide context for interpreting how different bibliometric indices may reflect contemporary academic activity.

Comparison of ih(5)-index rankings with cumulative h-index rankings revealed notable differences in institutional standing. Programs with historically strong productivity but relatively lower recent output maintained high cumulative h-indices yet demonstrated lower ih(5) scores. Conversely, some institutions ranked more favorably by ih(5) despite more modest cumulative citation counts. These findings highlight the distinction between legacy impact and recent scholarly activity. The ih(5)-index therefore provides a complementary perspective on institutional performance that may better reflect current research engagement and trajectory [15-18]. For residency applicants, faculty recruits, and institutional leaders, such temporal sensitivity may offer additional insight beyond cumulative indices alone.

Correlation analyses further support the conceptual validity of the ih(5)-index as a measure of recent productivity. The strong positive correlation (0.86) between ih(5) and total publications indicates that programs with higher recent productivity also tend to demonstrate greater overall output within the study window. Additionally, the moderate negative correlation between the Gini coefficient for publications and ih(5) (-0.40) suggests that programs with more evenly distributed scholarly productivity among faculty tend to have higher ih(5) scores. This finding may reflect the potential value of collaborative research environments in which scholarly activity is shared across a broader proportion of faculty rather than concentrated among a small subset of individuals. However, these associations represent correlations and should not be interpreted as causal relationships.

The relationship between ih(5)-index performance and faculty size further illustrates the complexity of institutional productivity. Larger programs may have access to broader subspecialty expertise, research infrastructure, and collaborative networks that could facilitate aggregate scholarly output. At the same time, departmental size alone does not necessarily correspond to sustained or equitably distributed productivity. Smaller programs may achieve competitive ih(5) scores through focused research agendas, strategic recruitment, or alignment of faculty interests. The observed associations between ih(5) and related indices such as ig(5), i10(5), and ie(5) indicate that programs performing well in one five-year bibliometric metric generally demonstrate strength across complementary measures, suggesting that these indices capture related dimensions of recent academic activity [4].

Finally, these findings should be interpreted within the broader context of neurosurgical research infrastructure. Prior studies have described variability in NIH funding among neurosurgeon-scientists and challenges in sustaining early-career research pathways [4,13,16]. Although the present study did not directly evaluate funding, protected research time, faculty composition, or collaboration networks, these factors may represent potential drivers of the institutional productivity patterns observed here. Future investigations linking bibliometric performance with measurable structural variables, such as grant funding levels, availability of protected research time, mentorship structures, faculty demographics, and collaborative publication networks, could further clarify the institutional factors associated with sustained academic productivity in neurosurgery.

Limitations

This study relies on publicly available data, which may include misattributed publications. Institutional variations in funding, faculty size, and resources also influence productivity but were not fully explored. Additionally, the ih(5)-index focuses on recent research output, which may underrepresent established faculty with fewer recent publications.

Another limitation is the reliance on citation metrics as a measure of research impact. Citations primarily reflect readership and scholarly engagement rather than the intrinsic quality of a study. Just as a well-executed study can generate citations, so too can a flawed study. Citation count and bibliometric measures therefore represent indicators of academic visibility and influence rather than direct assessments of scientific rigor or clinical relevance. Furthermore, factors such as self-citation, citation stacking, and preferential citation practices may introduce bias in bibliometric evaluations.

Lastly, our analysis does not capture qualitative aspects of scholarly contribution that are particularly relevant to neurosurgery. Factors such as mentorship of residents and fellows, leadership in surgical societies, contributions to guideline development, and involvement in surgical innovation are important components of academic influence but remain difficult to quantify using bibliometric approaches. Accordingly, the rankings presented in this study should be interpreted as reflecting patterns of recent scholarly activity rather than comprehensive measures of overall program quality or institutional excellence.

## Conclusions

This study presents a five-year bibliometric evaluation of all academic neurosurgical programs across the United States. The analysis identifies regional differences in both academic productivity and the distribution of scholarly output within departments. The ih(5)-index serves as a straightforward and interpretable metric that summarizes a department’s recent scholarly activity in neurosurgery by emphasizing productivity within a defined five-year window. By focusing on recent output, this index reduces the influence of historically prolific individuals and provides a clearer view of contemporary institutional research activity. Among the bibliometric measures evaluated, the ih(5)-index demonstrated the most consistent association with intradepartmental publication equality among the indices tested, with higher ih(5) values tending to reflect a more distributed pattern of scholarly productivity across faculty members. While this association does not imply causation, it suggests that recent institutional productivity may coincide with broader participation in research activity within departments.

By ranking institutions according to the ih(5)-index, this study highlights objective measures of recent academic output and provides a benchmark that may facilitate discussion about strategies to support scholarly activity within neurological surgery. Future work should focus on external validation of these findings, longitudinal reassessment of institutional productivity using repeated five-year windows, and investigation of how bibliometric performance relates to measurable structural factors such as research funding, training outcomes, and collaborative publication networks.
